# Anti-Cancer Properties of Resveratrol: A Focus on Its Impact on Mitochondrial Functions

**DOI:** 10.3390/antiox12122056

**Published:** 2023-11-29

**Authors:** Lolita Kursvietiene, Dalia M. Kopustinskiene, Inga Staneviciene, Ausra Mongirdiene, Kateřina Kubová, Ruta Masteikova, Jurga Bernatoniene

**Affiliations:** 1 Department of Biochemistry, Faculty of Medicine, Medical Academy, Lithuanian University of Health Sciences, Eiveniu str. 4, LT-50009 Kaunas, Lithuaniainga.staneviciene@lsmu.lt (I.S.); ausra.mongirdiene@lsmu.lt (A.M.); 2 Institute of Pharmaceutical Technologies, Faculty of Pharmacy, Medical Academy, Lithuanian University of Health Sciences, Sukileliu pr. 13, LT-50161 Kaunas, Lithuania; daliamarija.kopustinskiene@lsmuni.lt; 3 Department of Pharmaceutical Technology, Masaryk University, 60177 Brno, Czech Republic; kubovak@pharm.muni.cz (K.K.); masteikovar@pharm.muni.cz (R.M.); 4 Department of Drug Technology and Social Pharmacy, Faculty of Pharmacy, Medical Academy, Lithuanian University of Health Sciences, Sukileliu pr. 13, LT-50161 Kaunas, Lithuania

**Keywords:** resveratrol, cancer, mitochondria

## Abstract

Cancer is one of the most serious public health issues worldwide, demanding ongoing efforts to find novel therapeutic agents and approaches. Amid growing interest in the oncological applications of phytochemicals, particularly polyphenols, resveratrol—a naturally occurring polyphenolic stilbene derivative—has emerged as a candidate of interest. This review analyzes the pleiotropic anti-cancer effects of resveratrol, including its modulation of apoptotic pathways, cell cycle regulation, inflammation, angiogenesis, and metastasis, its interaction with cancer stem cells and the tumor microenvironment. The effects of resveratrol on mitochondrial functions, which are crucial to cancer development, are also discussed. Future research directions are identified, including the elucidation of specific molecular targets, to facilitate the clinical translation of resveratrol in cancer prevention and therapy.

## 1. Introduction

Cancer is characterized by the uncontrolled growth and spread of abnormal cells within the body [1]. Affecting people of all ages and backgrounds, it has a profound impact on society [2]. According to the World Health Organization, one in six deaths is caused by cancer, making it the second leading cause of death around the world [3]. Recent advancements in biomedical research have highlighted the role of phytochemicals as potential therapeutic anti-cancer agents [4,5,6]. Bioactive substances with diverse chemical structures and biological actions include phenols, flavonoids, quinones, coumarins, phenolic acids, tannins, terpenes, and alkaloids. Polyphenols, a subclass of phenolic chemicals, have a wide range of configurations, from monomeric forms with a single benzene ring to polymers with several aromatic rings [7,8].

Resveratrol, a polyphenolic stilbene derivative, which occurs naturally in grapes, red wine, and other plant sources, has gained significant interest due to its anti-neoplastic properties [9]. These properties involve a variety of cellular and molecular mechanisms, making resveratrol a potentially valuable candidate for both prevention and treatment of breast, prostate, lung, pancreatic, liver, colorectal, and other cancers. In addition, resveratrol exerts antioxidant, anti-inflammatory, and neuroprotective activity [8,10]. The anti-carcinogenic properties of resveratrol are facilitated by its diverse interactions with cellular signaling pathways implicated in several biological processes, such as apoptosis, control of the cell cycle, inflammation, angiogenesis, and metastasis [9]. Furthermore, it has been observed that resveratrol could restrict the expression of oncogenes, activate genes limiting tumor growth, and regulate the activity of transcription factors. Recent research has provided increasing evidence on the capacity of resveratrol to specifically target cancer stem cells, which frequently play a role in both resistance to traditional treatments and the recurrence of diseases [11]. Moreover, resveratrol can influence the tumor microenvironment, a crucial, yet sometimes disregarded element in the advancement of cancer and the response to treatment [12]. Thus, resveratrol shows promise in sensitizing cancer cells to established chemotherapeutic agents, offering potential synergistic effects, which may improve treatment efficacy and reduce side effects [9].

The anti-cancer properties of resveratrol are largely attributed to its impact on mitochondria—the organelles, which are commonly referred to as the “powerhouses” of the cell due to their crucial involvement in energy generation, cellular signaling, and programmed cell death, known as apoptosis [13]. These functions are intricately associated with the genesis and progression of cancer [14,15,16]. Resveratrol has a wide variety of effects on mitochondria, which presents an interesting opportunity for research on its potential therapeutic applications in the field of oncology [17]. The interactions, which include resveratrol and the activity of mitochondria offer a comprehensive strategy for the suppression of cancer, which can be used independently or in combination with other approaches [18]. Identifying specific molecular targets and elucidating the mechanisms of action of resveratrol are essential areas of ongoing research. These insights could facilitate the clinical use of resveratrol for chemoprevention, oncological therapies, and neuroprotection.

## 2. Chemical Properties and Sources of Resveratrol

Resveratrol belongs to a group of compounds called polyphenols. It has two phenolic rings connected by a styrene double bond. It is also known as 3,4′,5-trihydroxystilbene; hence, being a hydroxylated derivative of stilbene, it is attributed to the class of polyphenols known as stilbenes. Such chemical structure enables the formation of two isoforms, *trans* and *cis* isomers. The *trans* isomer is more stable and more common than *cis* isomer [19]. Poor resveratrol solubility in water (<0.05 mg/mL) affects its absorption and bioavailability [20].

In 1939, Takaoka successfully isolated resveratrol from the white hellebore plant (*Veratrum grandiflorum* O. Loes). Resveratrol is present in significant quantities in the dried roots of the plant *Polygonum cuspidatum* [21], as well as in grape berry skins and seeds, and it is particularly plentiful in red wine [22]. The concentration of resveratrol in grapes ranges from 0.16 to 3.54 μg/g [23]. Previous research has established that red wine has a much greater concentration of polyphenolic chemicals in comparison to white wine [23]. The concentration of resveratrol in different varieties of red wine exhibits a range of 0.1–15 mg/L [22]. Conversely, white wine demonstrates a concentration range of 0.1–2.1 mg/L [23]. Resveratrol can be found in a diverse range of plant species, including but not limited to pines, legumes, rhubarb, blueberries, raspberries, mulberries, and pistachios [22].

Resveratrol serves as an antioxidant in plants, protecting against photodamage. Furthermore, resveratrol is recognized as a phytoalexin. Plants synthesize such chemicals to protect them from stress induced by fungi, bacteria, or UV radiation [22]. *Trans* resveratrol is synthesized in *Vitis vinifera* grapes as a defense mechanism against the proliferation of fungal diseases, including *Botrytis cinerea* [24].

Food products often consist of a combination of the *cis* and *trans* isoforms of resveratrol, with the glycosylated form being the predominant variant. The prevalence of the *trans* isoform is higher in plant species [25]. Glycosylated resveratrol has enhanced stability and solubility; furthermore, glycosylation serves as a protective mechanism against oxidation [26,27]. In mammals, resveratrol undergoes rapid metabolism in the colon and liver [28]. Once in the plasma, it forms complexes with lipoproteins and albumin, thereby facilitating its cellular uptake [20].

## 3. Safety, Metabolism, and Pharmacokinetics of Resveratrol

Even at high concentrations (e.g., 1000–1500 mg/day), resveratrol is well tolerated by mammals [29]. As a dietary polyphenol, 100–1000 mg/day of resveratrol has been reported to be safe [19,30,31], confirmed in animal models in vivo [32] and clinical studies [33] as well. Although resveratrol at high doses up to 5 g has been reported to be non-toxic [34], in some clinical trials, resveratrol at daily doses of 2.5–5 g induced mild-to-moderate gastrointestinal symptoms [35] and diarrhea at twice-daily doses of 2 g [36]. It is important to note that resveratrol, which has been micronized, is more tolerable [37].

At least 70% of orally or intravenously administered 14C-labeled resveratrol is absorbed by humans [38]. Phase II metabolic enzymes (UDP-glucuronosyltransferases and sulfotransferases) are responsible for the metabolism in liver and intestinal microsomes. This pathway produces the well-known conjugates of resveratrol glucuronides and sulfates [31]. At low concentrations of resveratrol, glucuronides were the predominant metabolites, but at higher concentrations, there were more sulphates [39,40]. The metabolites dihydroresveratrol, 3,4′- dihydroxy-trans-stilbene, and lunularin are produced by human microbiota. In the circulation, low density lipoprotein and albumin transport resveratrol and its metabolites. Resveratrol enters cells through passive diffusion and can form complexes with integrin [41]. Studies implicated that complexation of resveratrol with serum proteins, fatty acids, lipoproteins, or integrins could reduce resveratrol accessibility to the cells [41]. After an oral dose of 25 mg in healthy human subjects, the concentrations of native resveratrol (40 nM) and total resveratrol (native resveratrol + resveratrol metabolites) (about 2 µM) in plasma suggested significantly greater bioavailability of resveratrol metabolites than native resveratrol [28,38]. The total plasma concentration of resveratrol did not exceed 10 µM following high oral doses of 2–5 g [36,37,42]. The bioavailability of micronized resveratrol (5 g/day) administered to colorectal cancer patients for 10–21 days was significantly greater than that of non-micronized resveratrol. The maximum serum concentration was threefold greater, at 8.51 µM and 2.40 µM, respectively, when using micronized resveratrol and non-micronized resveratrol [37,42].

Resveratrol is excreted in urine in its glucuronidated and sulfated forms. Interestingly, age and gender are known to influence resveratrol metabolism [43]. Several in vitro investigations demonstrated that the bioavailability of resveratrol can be increased by combining it with piperine or quercetin [19].

## 4. Pleiotropic Effects of Resveratrol

**Figure 1:**
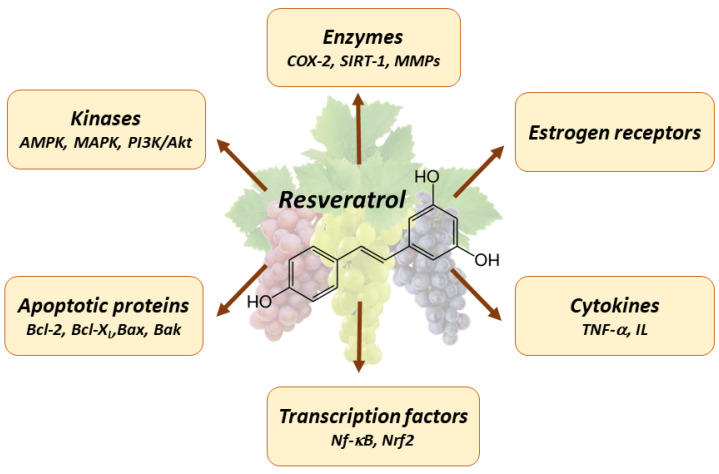
Main targets of resveratrol in tumor cells. COX-2—cyclooxygenase-2, SIRT-1—sirtuin 1, MMPs—matrix metalloproteinases, TNF-a—tumor necrosis factor alpha, IL—interleukins, NF-κB—nuclear factor kappa-light-chain-enhancer of activated B cells, Nrf2—nuclear factor erythroid 2–related factor 2, Bcl-2—B-cell lymphoma protein 2, Bcl-X_L_—Bcl-2 homologous splice variants, Bax—Bcl-2-associated X protein, Bak—Bcl-2 homologous antagonist killer 1, AMPK—AMP—activated protein kinase, MAPK—mitogen activated protein kinase, PI3K—phosphatidylinositide 3-kinases, Akt—protein kinase B.

Various research studies investigated the pleiotropic properties of resveratrol. Resveratrol has been found to demonstrate a variety of positive effects by influencing many molecular targets, including transcription factors, hormone receptors, caspases, cyclooxygenases, cyclins, sirtuins, interleukins, and others [23,44,45] (Figure 1). Resveratrol exerts anti-cancer activity through various molecular mechanisms, including the PI3K/Akt, STAT3/5, MAPK, AMPK/mTOR, SIRT1/NF-κB, and PGC-1α signaling pathways [46,47,48].

Numerous studies have provided evidence of the cardioprotective properties of resveratrol [49], its ability to inhibit platelet aggregation [50], its antioxidant effects [51], its anti-inflammatory properties [52], its capacity to lower blood glucose levels [53], its hepatoprotective effects [54], its neuroprotective properties [55], its potential in anti-aging interventions [54], and its anti-cancer activities [9,56]. A diet abundant in polyphenols has been found to be linked to a reduced risk of cardiovascular disease due to multiple factors. Resveratrol possesses the ability to impede platelet aggregation and promote vasodilation through the augmentation of nitrogen oxide generation [48,57]. Furthermore, it has been observed that resveratrol has the ability to decrease the oxidation of low density lipoproteins [58] and improve endothelial function [48].

Resveratrol exhibited protective effects against some neurodegenerative disorders, including Alzheimer’s disease [59], Parkinson’s disease [60], Huntington’s disease, and amyotrophic lateral sclerosis [61]. One of the neuroprotective mechanisms attributed to resveratrol involves the activation of sirtuins, which are histone deacetylases. This activation subsequently leads to the reduction in nuclear transcription factor NF-κB signaling [62]. This factor governs the regulation of many genes, which have also been associated with processes such as inflammation, cytoprotection, and carcinogenesis. Therefore, the anti-inflammatory actions of resveratrol involve many routes, which result in decreased NF-κB activation. In addition, it has been observed that resveratrol could impede the activity of cyclooxygenases—enzymes, which play a crucial role in the synthesis of pro-inflammatory mediators [63]. Furthermore, resveratrol could decrease the activity of microsomal prostaglandin E synthase-1—an enzyme, which is important in the creation of the pro-inflammatory prostaglandin PGE2 [64].

It is widely recognized that resveratrol has antioxidant properties at concentrations ranging from 5 to 10 μM. This is attributed to its ability to scavenge free radicals and enhance the activity of antioxidant enzymes. Moreover, the comparison of the anticarcinogenic characteristics of catechin, quercetin, gallic acid, and *trans* resveratrol demonstrated that resveratrol exhibited the greatest antioxidant activity among the aforementioned polyphenols after being administered twice a week for a duration of 18 weeks in the animal model [65]. Nevertheless, it has been observed that resveratrol exhibits pro-oxidant properties when present in doses ranging from 10 to 40 μM [23]. High concentrations of resveratrol can trigger apoptosis, suggesting its potential application in the field of cancer prevention [23,66].

## 5. Mechanisms of Resveratrol in Cancer Prevention

The ability of resveratrol to specifically alter numerous important cellular pathways implicated in tumor progression is one of the reasons it has gained so much attention as an anti-cancer agent with multiple applications [67,68,69,70,71]. A vast number of studies revealed the chemopreventive effect of resveratrol in various experimentally induced tumor models, such as skin, lung, gastric, colon, liver, pancreas, prostate, bladder, breast, ovarian, esophagus, thyroid, etc. [67,68,69,70,71]. Resveratrol exposure induced differentiation in canine oral mucosal melanoma cells, enhancing their sensitivity to cisplatin and increasing the mRNA expression of melanoma differentiation markers, such as microphthalmia-associated transcription factor (MITF) [72]. This differentiation effect was attributed to suppression of JNK signaling by resveratrol, as confirmed by inhibitor studies and decrease in JNK activity [72]. In estrogen receptor α-positive breast cancer cells, 17β-estradiol elevated neuroglobin levels, promoting cell survival [73]. Resveratrol could inhibit this pathway, but its rapid metabolism compromised efficacy; therefore, conjugating resveratrol with gold nanoparticles could enhance its bioactivity, offering potential in targeted breast cancer therapeutics [73]. Resveratrol sensitized colorectal cancer cells to 5-fluorouracil via β1-integrin receptors, modulating the tumor microenvironment and targeting the β1-integrin/HIF-1α signaling axis. This interaction could be important in the strategies to overcome 5-fluorouracil resistance in advanced colorectal cancer treatments [74].

**Figure 2:**
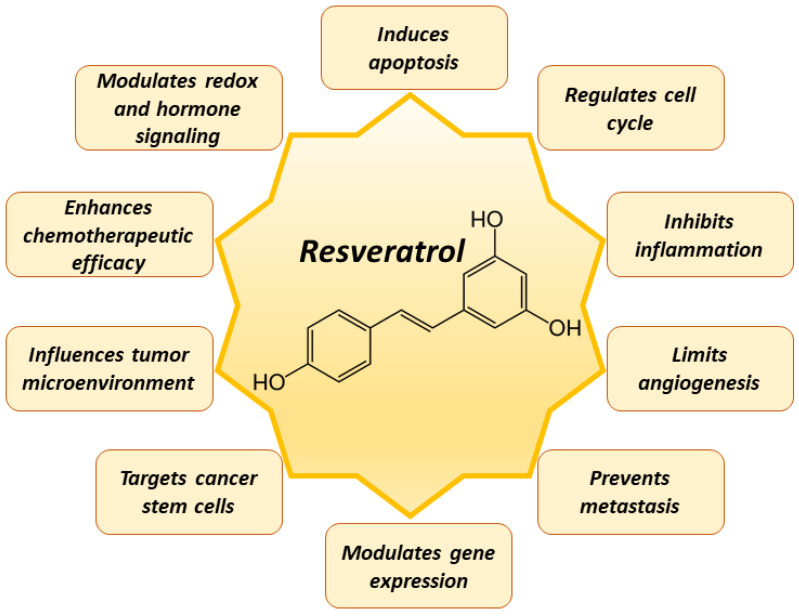
Main effects of resveratrol in tumor cells.

Resveratrol can influence a wide range of processes in tumor cells, including cell growth, apoptosis, stimulate transcription, hormone signaling, and inhibit tumor cell proliferation, inflammation, redox signaling, and angiogenesis [67,68,69,70,71] (Figure 2).

Resveratrol activates programmed cell death pathways in tumor cells. These effects are reviewed further in the subchapter “Mitochondria-Related Anti-Cancer Effects of Resveratrol”. Multiple molecular targets and signaling pathways are affected by resveratrol. It is known that resveratrol suppresses PI3-kinase, AKT, and NF-κB signaling pathways [75] and may affect tumor growth via other mechanisms as well. Many types of human cancer are associated with the changes in Akt signaling pathways [76]. Nuclear transcription factor NF-κB is known to be upregulated in many cancer cells, leading to tumor cell survival, proliferation, and metastasis formation [77,78]. The effects of resveratrol are linked to the suppression of NF-κB phosphorylation through SIRT1 signaling pathway [79,80].

Recent studies revealed that resveratrol induced autophagy and apoptosis by increasing the mRNA expression and activation of NGFR-AMPK-mTOR pathway in non-small-cell lung cancer A549 cells [75]. Protective autophagy was enhanced at resveratrol concentrations below 55 µM, while higher concentrations (above 55 µM) caused lethal autophagy [75]. It has been demonstrated that the proliferation of osteosarcoma cells in vitro and in vivo was reduced by resveratrol due to JAK/STAT3 pathway inhibition [11]. Phosphorylation of oncostatin M, JAK2, and STAT3 in MG-63 and MNNG/HOS cells decreased after resveratrol treatment for 48 h. Moreover, this study revealed that resveratrol abolished the self-renewal capacity of osteosarcoma cells [11].

Wnt signaling, a pathway activated in over 85% of colon cancers, was found to be suppressed in vitro and in vivo by resveratrol [81]. Resveratrol was shown to exert an inhibitory effect on the expression of β-catenins and also target genes c-Myc, MMP-7, and survivin in multiple myeloma cells, thus reducing the proliferation, migration, and invasion of cancer cells [82]. Resveratrol inhibited breast cancer stem-like cells in vitro and in vivo by suppressing Wnt/β-catenin signaling pathway, thus reducing cancerous cell population and inhibiting their proliferation [83].

In many types of cancer, the expression of Nrf2 was found to be elevated. Resveratrol activated the Nrf2 signaling pathway, causing separation of the Nrf2–Keap1 complex [84], leading to enhanced transcription of antioxidant enzymes, such as glutathione peroxidase-2 [85] and heme-oxygenase (HO-1) [86].

The effects of resveratrol on angiogenesis are related to the inhibition of both vascular endothelial growth factor (VEGF) and hypoxia-inducible factor (HIF)-1 generation, which leads to decreased secretion of VEGF [87]. The molecular mechanisms of resveratrol in acquired drug-resistant cancer cells have been summarized in a recent review [88]. Resveratrol was demonstrated to have an impact on drug bioavailability, regulation of cell cycle, DNA transcription, and autophagy. Moreover, it exerted pro-apoptotic and anti-metastatic activity [88]. Resveratrol could suppress leukemia cell proliferation and induce apoptosis due to increased expression of PTEN (the phosphatase and tensin homolog deleted on chromosome ten) and the inhibition of the activity of PI3K/AKT signaling pathway, resulting in decreased tumor cell proliferation, division, activated apoptosis, reduced angiogenesis, and formation of metastases [89]. Such multiple effects of resveratrol can be applied for prevention and treatment of various types of cancer [90].

Resveratrol enhances the sensitivity of cancer cells to chemotherapeutic agents through various mechanisms, such as promoting drug absorption by tumor cells, limiting drug metabolism by enzymes—such as cytochromes and glutathione-S-transferases—and reducing drug efflux [91]. In addition, resveratrol targets other resistance factors in cancer cells by influencing cell death pathways, including autophagy and apoptosis, adjusting reactive oxygen species (ROS) levels, modulating DNA repair processes, targeting cancer stem cells, and altering epigenetic factors, such as miRNAs [91]. Cancer stem cells are pivotal in tumor initiation and recurrence, with the tumor microenvironment components, such as cytokines, influencing cancer progression [92]. Resveratrol has been demonstrated to suppress tumor activity in lung cancer, specifically by inhibiting lung cancer stem-like cell stemness and reducing IL-6 levels [92]. Cancer-associated fibroblasts in the tumor microenvironment release cytokines, which promote tumor progression and the formation of cancer stem cells in oral cancer [93]. These fibroblasts, when stimulated, significantly increase the secretion of CXCL-12 and IL-6, which in turn boost cancer stem cell growth, proliferation, and metastatic potential [93]. Resveratrol nanoparticles have been shown to mitigate these effects by reducing the secretion of these cytokines, thus inhibiting cancer stem cell growth and other malignant behaviors [93]. One of the significant obstacles in cancer treatment is drug resistance, often leading to therapy failure and tumor relapse [94]. Resveratrol has demonstrated potential in addressing this issue by targeting cancer stem cells and non-coding RNAs, essential for stemness and drug resistance, thus impacting cancer initiation and the self-renewal of cancer stem cells [94].

All stages of carcinogenesis (initiation, promotion, and progression) are known to be affected by resveratrol. Resveratrol blocks initiation due to the activation of various carcinogen-detoxifying and antioxidant enzymes. It also suppresses promotion by inhibiting cyclooxygenase-2 activity [95], thus reducing prostaglandin synthesis and preventing DNA against oxidative damage. Moreover, resveratrol suppresses tumor progression due to induction of cancer cell cycle arrest, activation of apoptosis, and inhibition of angiogenesis and metastasis [71]. Due to its multifaceted effects on various phases of carcinogenesis, resveratrol is emerging as a potential anti-cancer agent. It functions by detoxifying carcinogens, providing antioxidant protection, inhibiting enzymes promoting cancer development, and protecting DNA from oxidative damage. With its broad spectrum of anti-cancer activities, resveratrol has the potential to be included in future cancer prevention and treatment strategies.

## 6. Chemotherapeutic Application of Resveratrol

The novel nano-formulations for resveratrol delivery include polymeric nanoparticles, liposomes, micelles, metallic nanoparticles, and solid lipid nanoparticles [96]. Various innovative resveratrol delivery systems, such as liposomes, micelles, polymeric nanoparticles [97], lipid-based nanocarriers [98], gold and silver nanoparticles [99], and silica nanoparticles [100], are known to improve the beneficial effects of resveratrol, including its anti-cancer efficacy. Such systems enable the improvement of several main properties of resveratrol, namely stability, solubility in water, penetration across biological membranes, and they provide enhanced permeation and access to cancer cells [96]. In order to increase the anti-cancer activity of resveratrol, it can also be used in nanomedicines in combination with various compounds or drugs, such as curcumin [101], quercetin [102], paclitaxel [103], docetaxel [104], 5-fluorouracil [105], and small interfering ribonucleic acids (siRNAs) [106,107].

An investigation of the effect of resveratrol in combination with common anti-cancer drugs docetaxel and doxorubicin in solid tumor cell lines MCF-7, HeLa, and HepG2 in vitro demonstrated that it increased the cytotoxicity of common chemotherapeutic drugs and alleviated the side effects, such as cardiac toxicity [108]. Increased expression of Bax and Bcl-2 was found when resveratrol was used in combination with docetaxel and doxorubicin [108]. Resveratrol could increase the anti-cancer effect of fluorouracil on murine hepatoma_22_ cells by inducing the S phase arrest of cancer cells [109]. Moreover, the toxicity of fluorouracil was markedly decreased by resveratrol [109].

The encapsulation of resveratrol in colloidal mesoporous silica nanoparticles significantly (approx. by 95%) enhanced both solubility and in vitro release kinetics of resveratrol [100]. The antibacterial and anti-cancer effect of resveratrol was elevated using both resveratrol-loaded gold and silver nanoparticles [99]. Recently, lecithin-encapsulated nano-resveratrol was demonstrated to be effective as an anti-cancer agent in vitro using human breast cancer cells BT474 [110].

Innovative delivery systems, such as polymeric nanoparticles, liposomes, micelles, and metallic nanoparticles, could enhance resveratrol stability, solubility, and ability to permeate biological membranes, ensuring more efficient access to cancer cells. When combined with other anti-cancer drugs, resveratrol has shown potential in increasing their cytotoxic effects and decreasing the associated side effects. Recent advancements, such as encapsulating resveratrol in colloidal mesoporous silica nanoparticles or lecithin, have further underscored its potential as a potent anti-cancer agent, especially when delivered in nano-formulations. However, the clinical studies on nano-resveratrol in various cancers (multiple myeloma, colon, liver, neuroendocrine tumor) have been summarized in a recently published review article [111]. There are currently insufficient clinical trials using nano-formulations of resveratrol. Most of the clinical trials initiated have not been completed yet.

## 7. The Role of Mitochondria in Cancer

**Figure 3:**
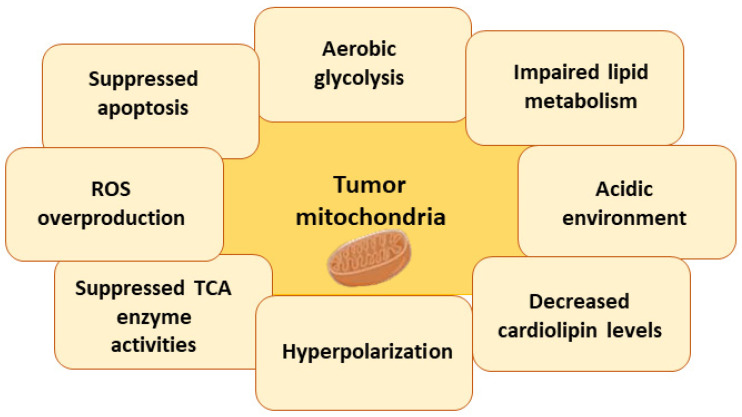
The main differences in tumor mitochondria compared to normal mitochondria.

The provision of a constant source of chemical energy, in the form of adenosine 5′-triphosphate (ATP), is important for the proper functioning of cells [112]. The inner membrane of mitochondria contains the electron transport chain (ETC), which serves as the primary enzymatic system responsible for the oxidation of carbohydrates, fats, and proteins. This process involves the conversion of the energy stored in the chemical bonds of reduced nicotinamide adenine dinucleotide (NADH) and reduced flavin adenine dinucleotide (FADH_2_) into ATP molecules [113]. Mitochondria are the primary location for adenosine triphosphate (ATP) synthesis in human cells. They are responsible not only for ATP synthesis but also for the biosynthesis of proteins, lipids, heme, iron–sulfur clusters. Moreover, mitochondria participate in apoptosis, maintenance of calcium and iron homeostasis, cell differentiation, induction of sirtuins, autophagy, ROS formation, and detoxification [114]. The disruption of at least one of these processes leads to mitochondrial-dysfunction-related aging, neurodegenerative disorders, cardiovascular diseases, inflammatory responses, and metabolic disorders [114,115]. The main changes in tumor mitochondria are shown in Figure 3.

### 7.1. Mitochondria, ROS, and Cancer

Mitochondria are the main organelles consuming oxygen in cells. Furthermore, they are also the main producer of ROS. Up to 2% of electrons flowing down in the electron transport chain (ETC) leak out under normal physiological conditions [116], react with oxygen, and form the superoxide anion radical O_2_^•−^, which is known as primary ROS. Then, other ROS, such as hydrogen peroxide H_2_O_2_ and hydroxy radical HO•, can be formed in the specific reactions (by superoxide dismutase and in the Haber–Weiss reaction, respectively). Mitochondria contain antioxidant enzymes, which are subject to regulation via certain signaling pathways. The activation of nuclear factor erythroid 2–related factor 2 (Nrf2) is a prominent mechanism in human cells, which contributes to antioxidant activity. This system controls the production of many antioxidant enzymes, including superoxide dismutase, catalase, glutathione peroxidase, and glutathione reductase [117,118]. Nrf2 additionally governs the modulation of enzymes included in phase II detoxification processes, such as glutathione-S-transferase, which plays a crucial role in the conjugation of xenobiotics or other harmful compounds with glutathione (GSH) [119]. When the mitochondrial antioxidant system is unable to detoxify ROS, a condition called oxidative stress develops. Mitochondrial inner membrane is the site of ROS production, but simultaneously, it is especially sensitive to lipid damage caused by ROS. Oxidative stress can increase the permeability of inner mitochondrial membrane, disrupt mitochondrial membrane potential, and impair the production of ATP. Moreover, elevated levels of ROS can induce mutations in mitochondrial DNA, which can lead to neurological, gastrointestinal, cardiac, respiratory, endocrinal, ophthalmological diseases in adults [120]. Thus, mitochondria are critically involved in the maintenance of the cellular redox balance—a process of extreme importance for various physiological functions.

The increased ROS generation in cancer cells presents a paradoxical situation [121]. On the one hand, ROS can be detrimental; they have the potential to induce cellular damage, primarily because of their high reactivity. They can target various cellular components, including proteins, lipids, and DNA. DNA damage inflicted by ROS can be especially dangerous for the cell, leading to mutations, some of which might provide a selective growth advantage, driving tumorigenesis and the progression of malignancies [121]. On the other hand, ROS are not merely destructive entities; they have evolved to serve significant physiological roles. In controlled amounts, ROS function as signaling molecules, regulating a plethora of cellular processes, from proliferation and differentiation to cellular responses to stress and inflammation [121]. They play a crucial role in maintaining cellular homeostasis. In cancer cells, the elevated ROS levels can modulate these signaling pathways, enhancing survival, growth and even metastatic spread of the tumor [121]. Furthermore, the influence of ROS extends beyond the cellular level. ROS can modulate the tumor microenvironment, influencing immune cell recruitment and function, thereby affecting the immune response against the tumor. Elevated ROS can also promote angiogenesis, facilitating the supply of nutrients to the rapidly growing tumor [121]. Thus, while the increased production of ROS in cancer cells can contribute to the genetic instability and DNA damage often associated with malignancies, ROS also play sophisticated roles in modulating cellular signaling and the tumor microenvironment [121]. Understanding the dual nature of ROS in cancer biology offers potential avenues for therapeutic strategies, either by targeting ROS directly or by manipulating their downstream effects.

### 7.2. Mitochondria, Apoptosis, and Cancer

Mitochondria play a crucial role in the regulation of apoptosis, often known as programmed cell death. In response to cellular stress, cells can release pro-apoptotic substances, such as cytochrome c [122]. This release subsequently triggers the activation of caspase enzymes, which are responsible for the dismantling of the cell. The process of oxidative stress can be intensified by an imbalance between the generation of reactive oxygen species (ROS) by mitochondria and the antioxidant defenses of the cell [122]. An increase in reactive oxygen species (ROS) has the potential to cause harm to cell functions, hence promoting apoptosis through disruption of the integrity of the mitochondrial membrane. Simultaneously, mitochondria serve as the location for anti-apoptotic proteins, which suppress these pro-death pathways, thus ensuring that cell death is strictly regulated and only occurs when necessary [122]. p53, commonly referred to as the “guardian of the genome”, is a tumor suppressor protein, which plays a crucial role in safeguarding against cancer [123,124]. It regulates cell health by pausing the cell cycle to facilitate DNA repair upon detecting damage and by initiating apoptosis to eliminate cells when damage is irreparable. Furthermore, p53 inhibits angiogenesis, which prevents tumors from developing the blood vessels they need to grow. In many cancers, a dysfunctional p53 pathway, resulting from mutations in either p53 or its regulatory components, impedes its role in apoptosis, thereby allowing damaged cells to proliferate uncontrollably [123,124].

Cancer cells are characterized by their inherent ability to undergo sustained proliferation, escape growth suppressors, and exhibit resistance to programmed cell death. To support these characteristics, they undergo profound metabolic changes, which differentiate them from their normal counterparts. The mitochondria, with their pivotal role in energy production, metabolism, and cell signaling, are at the forefront of these metabolic adaptations.

### 7.3. Metabolic Changes in Tumor Cell Mitochondria

#### 7.3.1. Warburg Effect

Tumor cells exhibit several distinct metabolic deviations compared to normal cells. One of the distinctive metabolic signatures of tumor cells is their increased dependence on aerobic glycolysis, commonly referred to as the Warburg effect, named after Otto Warburg, who first observed this phenomenon in the early 20th century [125]. While glycolysis is a process, which breaks down glucose to produce energy in the form of ATP, it typically operates in cells under low oxygen conditions. However, tumor cells, even in the presence of ample oxygen, predominantly utilize glycolysis over the more energy-efficient oxidative phosphorylation—a process, which healthy cells generally favor when oxygen is abundant [125]. Aerobic glycolysis allows these cells to rapidly produce ATP, which is crucial for their accelerated growth and proliferation. Moreover, the byproducts of glycolysis, including various metabolites, provide essential building blocks for the synthesis of nucleotides, lipids, and amino acids, supporting the biomass requirements of rapidly dividing cells [125]. While the Warburg effect may seem energetically inefficient compared to oxidative phosphorylation, it confers several advantages to tumor cells, enabling them to thrive and proliferate even in fluctuating oxygen environments and providing them with the metabolic flexibility to adapt and resist various therapeutic interventions.

#### 7.3.2. Impaired Lipid Metabolism

Cancer cells are characterized by significant disruptions in lipid metabolism. Specifically, these cells exhibit a pronounced inclination toward lipid biosynthesis driven by glutamine, an amino acid, altering typical cellular metabolic pathways [126,127]. This preference for glutamine-driven lipid biosynthesis in cancer cells serves multiple pivotal roles. First, it provides the necessary lipids, which are integral components of cell membranes, thereby supporting the structural and functional needs of rapidly dividing cells. Additionally, alterations in lipid metabolism contribute to the activation of several biochemical pathways implicated in both the initiation of cancer (tumorigenesis) and the process whereby cancer spreads to other parts of the body (metastasis) [126,127]. These pathways, enhanced by the aberrant lipid metabolism, enable cancer cells to modulate their environment, resist apoptotic signals, and invade distant tissues, rendering them more aggressive and resilient to conventional therapeutic strategies. Moreover, the products of altered lipid metabolism can also serve as signaling molecules and modulators of gene expression, further reinforcing the aggressive and adaptable nature of cancer cells [126,127]. These modifications in lipid metabolic pathways offer insights into the intricate metabolic reprogramming of cancer cells and represent potential targets for therapeutic intervention to impair cancer progression.

#### 7.3.3. Acidic Environment

Tumor cells exhibit a distinct metabolic phenotype, which significantly regulates both intracellular and extracellular pH levels, resulting in impaired pH balance. The intracellular processes—predominantly the elevated rate of glycolysis often present in cancer cells—lead to increased production of lactic acid, subsequently causing a reduction in the pH of the extracellular environment, making it more acidic [128]. This acidic microenvironment serves to promote inflammation. The low pH condition triggers the release of pro-inflammatory cytokines and chemokines, leading to the recruitment of inflammatory cells. This inflammation within the tumor microenvironment is a well-recognized hallmark of cancer, often associated with enhanced tumor growth, progression, and metastasis. The acidic extracellular environment promotes the activity of enzymes, such as cathepsins and matrix metalloproteinases, which destroy the extracellular matrix, allowing cancer cells to invade the surrounding tissues [129]. It also impacts the surrounding non-tumorous cells, modifying their function and potentially inducing a pro-tumoral phenotype, thereby creating a supportive niche for tumor progression.

#### 7.3.4. Changes in Cardiolipin Levels and Impaired Activity of Mitochondrial Enzymes

Mitochondrial enzyme activity is often impaired in tumor cells, resulting in changes in metabolism and energy balance [14,130]. Central to these alterations is the lipid cardiolipin—the main phospholipid of the inner mitochondrial membrane. Cardiolipin is essential for maintaining the integrity of the mitochondrial membrane and plays a vital role in anchoring and stabilizing many mitochondrial proteins and enzymes. In cancer cells, alterations in cardiolipin levels and composition have been observed, and these changes can have profound implications for mitochondrial function [131,132,133]. When cardiolipin levels are disrupted, this can affect the optimal functioning of several mitochondrial enzymes, including those involved in the electron transport chain, which is crucial for oxidative phosphorylation [131,132,133]. The changes in enzymatic activity can lead to reduced efficiency in energy production and can also increase ROS production. An elevation in ROS can further induce oxidative stress, damage the cellular components, and modulate the signaling pathways, which can promote tumorigenesis [121,131,132,133]. Moreover, the compromised enzyme activities in the mitochondria of tumor cells can also alter other metabolic pathways, such as the Krebs cycle, fatty acid oxidation, and amino acid metabolism [131,132,133]. Such disruptions can force the cell to rely more on alternative metabolic routes, such as glycolysis, even in the presence of oxygen, further underscoring the metabolic flexibility and adaptability of cancer cells.

#### 7.3.5. Hyperpolarization of Mitochondria

Mitochondria maintain a voltage difference across their inner membrane, known as the mitochondrial membrane potential (ΔΨ_m_). This potential is crucial for numerous mitochondrial functions, primarily the production of ATP through oxidative phosphorylation. In cancer cells, an intriguing alteration observed is the hyperpolarization of their mitochondria [14]. This condition arises due to the more significant accumulation of protons in the intermembrane space and a corresponding more negative charge inside the mitochondrial matrix. The various causes of this hyperpolarization in cancer cells include altered metabolic pathways, changes in electron transport chain efficiency, or even changes in the activities of ion channels and transporters [14]. Mitochondrial hyperpolarization indicates that the mitochondria in these cells might be working at a higher energetic level, potentially driving increased ATP synthesis, which could satisfy the increased energy demands of rapidly proliferating cancer cells. Studies have suggested that the hyperpolarized state of mitochondria is directly proportional to the aggressive nature of the cells [14]. The more hyperpolarized the mitochondria, the higher the potential of the cancer cell for invasiveness, metastatic spread, and resistance to therapies [14]. Moreover, this hyperpolarized phenotype might play roles in cellular signaling, modulating the pathways associated with cell survival, growth, and even immune evasion.

Thus, the growth, survival, and aggression of tumor cells depend on mitochondria-driven metabolic changes. Understanding these alterations provides insights into potential therapeutic targets and strategies for cancer treatment.

## 8. Mitochondria-Related Anti-Cancer Effects of Resveratrol

**Figure 4:**
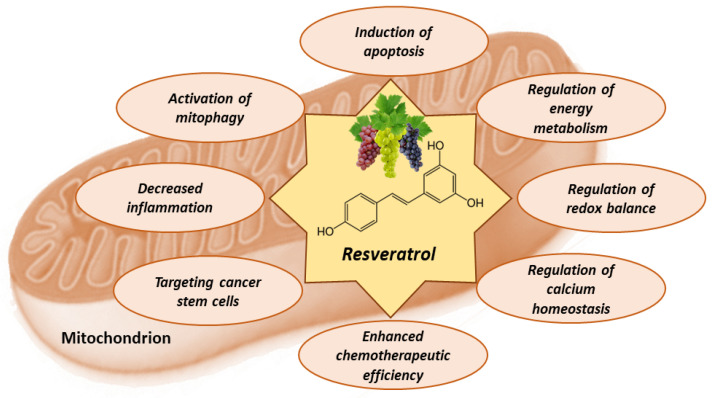
The effects of resveratrol on mitochondrial functions in tumor cells.

Resveratrol exhibits various anti-cancer properties, including the initiation of apoptosis through mitochondrial pathways, interference with energy metabolism in cancer cells, and enhancing the oxidative stress within the mitochondria of these cells, leading to cell damage and death. Additionally, it can modulate mitochondrial calcium, increase the efficacy of existing chemotherapeutic agents, target cancer stem cells, alleviate inflammation associated with tumors, and promote the elimination of impaired mitochondria (Figure 4).

The effects of resveratrol on mitochondria are complex and depend on various factors, including cell type and physiological context (Table 1).

### 8.1. Resveratrol Activates Apoptosis in Tumor Cells

Resveratrol has been shown to induce mitochondria-mediated apoptosis in cancer cells [147,148]. This is significant, as apoptosis is often dysregulated in cancer, allowing for uncontrolled cell proliferation. The interaction of resveratrol with the mitochondria can lead to the release of cytochrome c, a critical component in the initiation of the apoptotic cascade [147,148]. Resveratrol activates pro-apoptotic proteins, such as Bax and Bak. Once activated, these proteins can permeabilize the mitochondrial outer membrane, leading to the release of apoptogenic factors, including cytochrome c [147,148]. In addition to activating pro-apoptotic proteins, resveratrol also inhibits anti-apoptotic proteins, such as Bcl-2 and Bcl-xL. Through the inhibition of these proteins, resveratrol has the capacity to shift the equilibrium in favor of apoptosis [147,148]. Studies have demonstrated that resveratrol could amplify oxidative stress within mitochondria, leading to mitochondrial dysfunction and further promoting apoptosis [147,148]. One of the early events in the induction of apoptosis is the loss of mitochondrial membrane potential. Resveratrol can induce its decrease, making the cell more susceptible to the apoptotic process [147,148]. The effects of resveratrol are not only limited to direct interactions with mitochondrial proteins. It can also influence various cellular signaling pathways, which can ultimately impact mitochondrial function and the induction of apoptosis, leading to tumor cell death [147,148]. In the investigations of the impact of resveratrol (2.5–100 μM for 48 h) on mouse neuroblastoma cells Neuro-2a and NB41A3, cytotoxic effects were observed, where apoptosis and autophagy were identified as the predominant mechanisms of cell death, as opposed to necrosis [134]. Resveratrol induced the production of Grp78 protein and ROS in neuroblastoma cells in a time-dependent manner. Furthermore, the suppression of the ER-stress–ROS signaling axis resulted in a considerable reduction in resveratrol-induced autophagy, DNA damage, and cell death [134]. Additionally, resveratrol decreased phosphorylation of the retinoblastoma protein, leading to the arrest of the cell cycle at the S phase, and facilitated the translocation of the Bak protein to the mitochondria, resulting in a drop in the mitochondrial membrane potential [134]. This event subsequently triggered the activation of caspases-9, -3, and -6, ultimately leading to DNA fragmentation [134]. Another study examined the anti-cancer effects and mechanism of resveratrol (2–500 µg/mL) in human colorectal cancer cells HCT116 and SW620 [135]. The results demonstrated that resveratrol dose-dependently reduced colorectal cancer cell viability and enhanced apoptosis and ROS levels relative to the control group [135]. In resveratrol-treated colorectal cancer cells, Bax, cytochrome c, cleaved caspase-9, and cleaved caspase-3 were increased, while Bcl-2 was downregulated relative to control cells [135]. Resveratrol’s anti-viability effects were compared among various human cancer cell types, showing that resveratrol (1–100 µM for 24 and 48 h), starting from 10 µM and in a time-dependent manner, markedly suppressed cell viability in U937 and MOLT-4 leukemia cells, moderately inhibited it in MCF-7 breast, HepG2 liver, and A549 lung cancer cells, and slightly inhibited it in Caco-2, HCT116, and SW480 colon cancer cells [136]. U937 and MOLT-4 showed a significant increase in late apoptosis after resveratrol treatment, while MCF-7 and HepG2 showed an increase in early apoptosis, whereas only leukemic cells showed DNA fragmentation [136]. Sirtuin 1 and adenosine-monophosphate-activated protein kinase was not responsible for apoptosis, stimulated by resveratrol [136]. Resveratrol decreased Akt activation and H-Ras, facilitating Bax translocation to mitochondria in leukemic cells [136]. Resveratrol demonstrated differential pro-apoptotic effects in colorectal cancer through modulation of Sirt-1 and p53 [149]. Higher concentrations suppress Sirt-1, enhancing p53 acetylation, and apoptosis in wild-type colorectal cancer cells, suggesting a novel negative regulatory interplay between p53 and Sirt-1 in resveratrol-treated colorectal cancer [149]. Thus, by inducing apoptosis, resveratrol can suppress tumor growth, making it a compound of interest for therapeutic applications in oncology.

### 8.2. Resveratrol Counteracts Warburg Effect

Resveratrol has a modulatory effect on cellular energy metabolism—another critical function of mitochondria. Cancer cells often shift their metabolic focus toward glycolysis, even in the presence of oxygen—a phenomenon known as the Warburg effect [150]. Resveratrol inhibits aerobic glycolysis, thereby reducing cancer cell survival and proliferation [151]. Resveratrol modulated glucose metabolism in various types of cancer, including breast, prostate, lung, pancreatic, liver, colorectal, and others [138,152,153,154,155]. Cancer cells have increased glucose absorption due to overexpression of Glut transporters and are characterized by lactate fermentation even under aerobic conditions [150]. An excess of lactate causes increased acidity (low pH levels) in cancer cells [150]. The main regulatory enzymes of glycolysis, such as hexokinase-2, phosphofructokinase, aldolase, glucose-6-phosphate dehydrogenase, enolase, pyruvate kinaseM2, are known to be overexpressed in cancer cells [151]. Uncontrolled proliferation of cancer cells requires a large amount of energy and sufficient supply of nucleotides for DNA synthesis. Increased uptake of glucose and overexpression of enzyme glucose-6-phosphate dehydrogenase in cancer cells enable attaining sufficient ribose-5-phosphate and the reducing equivalents in the form of NADPH in the pentose phosphate pathway [151]. Ribose-5-phosphate and NADPH are vital for cancer cell survival because they are necessary for de novo nucleotide synthesis [151]. Resveratrol can modify glucose/carbohydrate metabolism in cancer cells by affecting several signaling pathways, the activity of some transcription factors, as well as gene expression. It has been demonstrated in several studies that resveratrol inhibits glycolysis through the PI3K/Akt/mTOR signaling pathway in human cancer cells [140,141]. In addition, resveratrol was reported to inhibit the growth of cells through metabolic shifting from aerobic glycolysis to oxidative phosphorylation in PC3 prostate cancer cells [146]. Moreover, resveratrol reduced glucose uptake by cancer cells due to targeting carrier Glut1; therefore, less lactate was produced, and consequently, the survival of tumor cells was reduced as well [151]. Resveratrol (100 µM for 48–72 h) had a negative impact on hexokinase II (HK2)-mediated glycolysis, leading to a significant inhibition of both anchorage-dependent and -independent growth in non-small-cell lung cancer cells [139]. Moreover, the activation of EGFR and downstream kinases Akt and ERK1/2 was observed to diminish upon exposure to resveratrol [139]. Resveratrol (10–150 µM for 24 h), in a concentration-dependent manner, inhibited glucose absorption in Lewis lung carcinoma, HT-29 colon, and T47D breast cancer cells via modulation of the ROS-driven activation of hypoxia-inducible factor-1 [138]. Resveratrol also suppressed colon cancer cell HCT116 and Caco-2 proliferation with half maximum inhibitory concentrations (IC_50_ of 50 and 130 µM) for HCT116 and Caco-2, respectively [137]. Caco-2 cells showed a significant time-dependent increase in the glycolytic pathway, whereas HCT116 cells did not. Resveratrol (100 µM for 48–72 h) inhibited glycolytic enzymes (pyruvate kinase and lactate dehydrogenase) in Caco-2 cells while increasing citrate synthase activity and decreasing glucose consumption in both cell lines [137]. Moreover, resveratrol suppressed the expression of leptin and c-Myc while increasing the level of vascular endothelial growth factor. Caspases 3 and 8, which are apoptotic indicators, were activated, and the Bax/Bcl2 ratio was raised [137].

Resveratrol can also influence the electron transport chain in mitochondria, potentially limiting ATP production in cancer cells, leading to suppressed tumor proliferation [148]. It was reported that resveratrol could inhibit mitochondrial ATP synthase with IC_50_ 19 µM [156]. The deprivation of ATP disrupts critical processes within tumor cells, as they rely heavily on a constant energy supply to maintain their rapid growth and replication [148]. Counteracting the Warburg effect and restricting ATP synthesis, resveratrol could potentially enhance the efficacy of cancer therapies by rendering tumor cells more susceptible to treatment interventions.

### 8.3. Antioxidant and Pro-Oxidant Effects of Resveratrol

The influence of resveratrol on mitochondrial oxidative stress appears to be selectively detrimental to cancer cells. By amplifying the oxidative stress within the mitochondria of these cells, resveratrol induces a state of cellular toxicity, which leads to damage and subsequent cell death while often sparing healthy cells [17,143,157]. Resveratrol displays a dual behavior concerning ROS balance: it acts as an antioxidant in regular conditions but as a strong pro-oxidant in cancer cells, initiating apoptosis pathways. Both antioxidant and pro-oxidant activities are involved in the anti-cancer effects of resveratrol [17,143,157]. Investigations have demonstrated that HeLa cells exposed to resveratrol for 24 h exhibited concentration-dependent increases in cell growth inhibition, ROS activation, and light chain 3-II expression [142]. Intriguingly, HeLa cells exposed to resveratrol for 30 min exhibited a concentration-dependent effect, with low concentrations of resveratrol (25 µmol/L) reducing ROS level, inhibiting transcription and expression levels of light chain 3-II expression, and stimulating mitochondrial respiratory capacities [142]. In contrast, high concentrations of resveratrol (50 and 100 µmol/L) induced ROS overproduction and autophagy in the cells, resulting in decreased mitochondrial membrane potential, DNA copy numbers, and respiratory capacities [142]. Resveratrol was tested for its effect on cancer cell proliferation using HeLa, MDA-MB-23, MCF-7, SiHa, A549, HUVEC, and 3T3 cell lines [143]. After 48 h, resveratrol exhibited greater effectiveness in inhibiting metastatic HeLa and MDA-MB-23 (IC_50_ = 200–250 μM) cells compared to low metastatic MCF-7, SiHa, and A549 (IC_50_ = 400–500 μM) and non-cancer HUVEC and 3T3 (IC_50_ ≥ 600 μM) [143]. In HeLa cells, resveratrol (200 μM/48 h) dramatically reduced OxPhos protein concentrations (30–90%) and fluxes (40–70%) compared to non-treated cells [143]. Using succinate as an oxidizable substrate, resveratrol (100 μM/1–5 min) significantly reduced the OxPhos flux (net ADP-stimulated respiration) in isolated tumor mitochondria (>50%) compared to non-tumor mitochondria (<50%) [143]. Resveratrol also increased cellular ROS production (2–3 times) and decreased SOD activity (but not content) and GSH levels, while catalase, glutathione reductase, glutathione peroxidase, and glutathione-S-transferase activities remained unchanged [143]. Furthermore, resveratrol (200 μM/48 h) caused cellular death through high mitophagy activation (65%), not apoptosis [143]; it also stimulated ROS overproduction, which inhibited OxPhos and stopped metastatic HeLa cancer cell proliferation [143]. Thus, resveratrol selectively upregulates oxidative stress within mitochondria, causing damage and death to cancer cells. The potential of resveratrol to maintain the integrity of normal cells and trigger programmed cell death in tumor cells renders it an attractive candidate for cancer treatment strategies.

### 8.4. Effects of Resveratrol on Calcium Homeostasis

Resveratrol, by interacting with mitochondrial calcium channels, can influence the intracellular calcium levels, thus impacting the homeostasis and metabolic activity of the cells [66,148,158]. Changes in mitochondrial calcium can have downstream effects on cellular processes, including energy metabolism and apoptosis, which are crucial for the survival and growth of cancer cells [66,148,158]. Increased calcium levels in the mitochondria can activate various enzymes and alter the mitochondrial membrane potential, affecting cell functions and overall viability [148]. Changes in mitochondrial calcium uptake due to resveratrol can influence multiple cellular signaling pathways, which are integral in regulating cellular functions, such as cell cycle progression, cellular differentiation, and cellular response to stress [148]. These pathways play a critical role in determining the fate of tumor cells—whether they will continue to proliferate or undergo programmed cell death. Additionally, the modulation of these pathways by resveratrol may contribute to the disruption of the supportive tumor microenvironment and hinder the establishment and progression of tumors [66,148,158]. By affecting the calcium dynamics within the mitochondria, resveratrol can induce stress in cancer cells, disrupt their metabolic adaptability, and potentially make them more susceptible to anti-cancer therapies [148]. Investigations of the effects of resveratrol (100 μM/36 h) on cell viability, caspase activity, calcium levels in various cell components, ATP content, and mitochondria–ER junctions in cell lines EA.hy926 and HeLa and HUVEC somatic cells revealed that resveratrol induced cell death selectively in cancer cells by enhancing mitochondrial calcium uptake [144]. The anti-cancer effect of resveratrol (20–100 μg/mL for 24 h) and the function of sodium/lithium/calcium exchanger in relation to calcium ions were investigated in HeLa cells [145]. This therapeutic approach, employing siNCLX-mediated gene silencing and drug therapy with resveratrol, demonstrated a disruption of calcium homeostasis, an increase in caspase (3, 8, 9) mRNA expressions, and DNA damage, all of which resulted in apoptotic cell death [145]. Ca^2+^ overload killed HeLa cells in response to the inhibition of mitochondrial Ca^2+^ extrusion together with resveratrol therapy [145]. Thus, resveratrol influences mitochondrial calcium channels, thereby affecting intracellular calcium levels and cellular processes, such as energy metabolism, apoptosis, and multiple signaling pathways, essential for cancer cell survival and proliferation. This modification can induce stress in cancer cells, impair their metabolic adaptability, and increase their susceptibility to anti-cancer treatments.

### 8.5. Effects of Resveratrol on Metabolic Plasticity, Cancer Stem Cells, and Tumor Microenvironment

Metabolic plasticity and the ability to evade harmful effects of chemotherapeutic medicines are crucial for tumor cell survival. The process relies heavily on mitochondria, which control energy production, calcium homeostasis, and apoptotic pathways [159,160]. The mitochondrial effects of resveratrol can lead to metabolic and functional alterations, which weaken the resistance of tumor cells to chemotherapy. For example, resveratrol can induce apoptosis in tumor cells by changing the mitochondrial membrane potential or by shifting the balance between pro-apoptotic and anti-apoptotic proteins [159,160]. Standard chemotherapeutic medicines can have a greater cytotoxic effect when combined with resveratrol. Because of this synergistic impact, it is possible that standard drug dosages can be decreased while maintaining or even improving therapeutic outcomes [159,160].

Cancer stem cells are a unique subset of cells within a tumor, which possess the ability to self-renew and drive tumor growth [17]. Due to their inherent resistance mechanisms, these cells often escape conventional cancer treatments, leading to relapse and metastasis [17,161]. Unlike differentiated cancer cells, cancer stem cells show a distinct metabolic profile characterized by a heightened reliance on mitochondrial oxidative phosphorylation for energy production [17,161]. This makes their mitochondrial function a potential target for therapeutic interventions. Preliminary studies have hinted at the ability of resveratrol to target the mitochondrial metabolism of cancer stem cells. By inhibiting the bioenergetic function of mitochondria in these cells, resveratrol may deplete their energy reserves, thereby hampering their survival and proliferation [162]. While the exact mechanisms remain to be fully elucidated, it is postulated that resveratrol disrupts the electron transport chain in the mitochondria of cancer stem cells, leading to a reduction in ATP production [162]. This energy crisis in cancer stem cells may activate cellular stress pathways and promote cell death [162]. The ability of resveratrol to target the mitochondrial bioenergetics of cancer stem cells presents a novel approach for eliminating this challenging cell population. By specifically targeting cancer stem cells, resveratrol may enhance the efficacy of standard cancer treatments and reduce the chances of tumor relapse [17,161].

The tumor microenvironment, which comprises cancer cells, stromal cells, and the extracellular matrix, often exhibits a pro-inflammatory state [12,17]. This inflammatory microenvironment is characterized by the release of cytokines, chemokines, and growth factors, which promote tumor growth, angiogenesis, and metastasis [12,17]. Chronic inflammation in this setting not only supports tumor progression but also helps in evading the immune system. The interaction of resveratrol with mitochondrial pathways plays a pivotal role in its anti-inflammatory effects [12,17]. By modulating mitochondrial functions, resveratrol can influence the production of ROS and other inflammatory mediators [12,17]. The reduction in ROS levels and the subsequent decrease in oxidative stress can lead to a reduction in the activation of various pro-inflammatory pathways. Resveratrol has been shown to downregulate the production of pro-inflammatory cytokines, such as TNF-α, IL-6, and IL-1β [87]. By inhibiting these cytokines, resveratrol can disrupt the signals promoting inflammation within the tumor environment. The mitigation of the inflammatory milieu within tumors can potentially hinder tumor growth and progression. A less inflammatory tumor environment might be less favorable to angiogenesis, tissue invasion, and metastasis [87]. Furthermore, by reducing inflammation, resveratrol might also restore the function of immune cells within the tumor, making them more effective in targeting and eliminating cancer cells.

### 8.6. Effects of Resveratrol on Mitophagy

Mitophagy, a specialized form of autophagy, plays a central role in maintaining cellular homeostasis by selectively degrading damaged mitochondria. By eliminating malfunctioning mitochondria, mitophagy prevents the accumulation of ROS and safeguards the cell from detrimental metabolic consequences [143]. Dysregulated mitophagy has been implicated in various diseases, including cancer. Inefficient removal of damaged mitochondria can result in heightened oxidative stress, which can lead to DNA damage, mutations, and eventually, tumorigenesis [163,164,165]. Furthermore, some cancer cells may exploit mitophagy for survival, especially under metabolic stress or therapeutic interventions. Resveratrol has been shown to activate mitophagy pathways [163,164,165]. By upregulating certain mitophagy-related proteins and receptors, resveratrol ensures the swift identification and removal of damaged mitochondria from the cell. This action can minimize the harmful effects of mitochondrial dysfunction, such as oxidative damage, which might otherwise promote carcinogenesis [163,164,165]. One of the ways in which resveratrol promotes mitophagy is through the activation of the protein SIRT1, a known regulator of autophagy and cellular health [166]. Once activated, SIRT1 can stimulate the deacetylation and subsequent activation of essential mitophagy players, thereby promoting the encapsulation and degradation of impaired mitochondria [166]. The ability of resveratrol to improve mitophagy offers a dual advantage in cancer management. On the one hand, it prevents the accumulation of damaged mitochondria, which can drive cancer progression, and on the other, it can make cancer cells more vulnerable to therapies by denying them the survival benefits of mitophagy under stress.

## 9. Conclusions and Future Perspectives

The multifaceted effects of resveratrol in cancer management point to its potential role as a versatile tool in oncological interventions. The ability of resveratrol to regulate cellular pathways, interfere with mitochondrial functions in cancer cells, and exert significant effects on cellular metabolism, apoptosis, and energy generation indicates a profound importance in the field of cancer therapies. To overcome the limits of current treatments and improve patient outcomes, groundbreaking research is targeted at utilizing the capacity of resveratrol to synergize with existing chemotherapeutic drugs. The incorporation of advanced technologies, such as nanotechnology, is critical for improving the distribution and specificity of resveratrol and addressing current bioavailability issues. Furthermore, the discovery of resveratrol analogs could enhance treatment efficacy and overcome the adaptive resistance of tumor cells. The growing interest in creating preventive measures highlights the involvement of resveratrol in preventing cancer growth and altering the tumor microenvironment, especially in colon and breast cancers. As more conclusive clinical evidence becomes available and knowledge from other scientific fields is integrated, resveratrol is likely to become an integral part of future cancer therapies and preventative measures.

## Figures and Tables

**Table 1:** Main mitochondria-related effects of resveratrol on different cancer types.

Cancer Type	Model Used	Experimental Conditions	Effect of Resveratrol	Ref.
Neuroendocrine cancer	Mouse neuroblastoma cells Neuro-2a and NB41A3	2.5–100 μM/48 h	Induction of apoptosis	[134]
Colorectal cancer	Human HCT116 and SW620	2–500 µg/mL	Decreased cell viability, enhanced apoptosis, increased ROS level	[135]
	Human Caco-2, HCT116, and SW480	10–100 µM/24 and 48 h	Decreased cell viability	[136]
	Human HCT116 and Caco-2	100 µM/48–72 h	Suppressed proliferation, inhibited glycolytic enzymes	[137]
	HT-29	10–150 µM/24 h	Suppressed glucose uptake	[138]
Leukemia	Human U937 and MOLT-4	10–100 µM/24 and 48 h	Decreased cell viability, DNA fragmentation	[136]
Breast cancer	Human MCF-7	10–100 µM/24 and 48 h	Decreased cell viability, enhanced apoptosis	[136]
	T47D	10–150 µM/24 h	Suppressed glucose uptake	[138]
Liver cancer	Human HepG2	10–100 µM/24 and 48 h	Decreased cell viability, enhanced apoptosis	[136]
Lung cancer	Human A549	10–100 µM/24 and 48 h	Decreased cell viability	[136]
	Human NSCLC cells	100 µM/48–72 h	Inhibited hexokinase II (HK2)-mediated glycolysis	[139]
	Lewis lung carcinoma	10–150 µM/24 h	Suppressed glucose uptake	[138]
B-cell carcinoma	Human GC-like DLBCL cell lines	25 or 50 μM/24 h	Glycolysis inhibition	[140]
Ovarian cancer	Ovarian cancer cells	50 μM/48 h	Glycolysis inhibition	[141]
Cervical cancer	HeLa	25 µmol/L/30 min	Decreased ROS level, stimulated mitochondrial respiration	[142]
		50 and 100 µmol/L/30 min	Increased ROS level, stimulated autophagy	[142]
		200 μM/48 h	Induced mitophagy and ROS overproduction	[143]
		100 μM/36 h	Increased calcium uptake and induced apoptosis	[144]
		20–100 μg/mL/24 h	Induced calcium overload and apoptosis	[145]
Prostate cancer	Human PC3	10 µM/48 h	Enhancement of oxidative phosphorylation	[146]
